# Correction: Perception of the quality of remote lessons in the time of COVID-19: A comparative study in Latin America quality of remote lessons in the context of COVID-19

**DOI:** 10.1371/journal.pone.0314587

**Published:** 2024-11-21

**Authors:** Lida Esperanza Villa Castaño, William Fernando Durán, Paula Andrea Arohuanca Percca

The subtitle “quality of remote lessons in the context of COVID-19” is incorrectly included in the title. The correct title is: Perception of the quality of remote lessons in the time of COVID-19: A comparative study in Latin America. The correct citation is: Villa Castaño LE, Durán WF, Arohuanca Percca PA (2022) Perception of the quality of remote lessons in the time of COVID-19: A comparative study in Latin America. PLOS ONE 17(6): e0268966. https://doi.org/10.1371/journal.pone.0268966.
