# Supplementary material for: Perception of the quality of remote lessons in the time of COVID-19: A comparative study in Latin America quality of remote lessons in the context of COVID-19
Source: PLoS One. 2022 Jun 3;17(6):e0268966. doi: 10.1371/journal.pone.0268966 (PMC9165895; doi:10.1371/journal.pone.0268966)
Supplement: Attachment — Submitted filename: comments.docx [file pone.0268966.s001.docx]

Thank you for the opportunity to read your valuable article.

Introduction

1)This is a valuable study that investigated students' perceptions of quality in the pandemic. There could be a pandemic going forward. Research results may be a measure of student status during a pandemic, rather than in peacetime. The clearer the extraction process for the question items, the more reliable it is.

Introduction, an overview of this study is included, but there is no presentation of the purpose of the research.

Method

2) The analysis method is reasonable. However, the method of qualitative analysis, which is the basis of this study, is unclear.

It seems that there is a lack of analytical methods for parts qualitatively.

3) Why did only students from the area participate in Group Focus Interview (GFI)?　How did the GIF progress?

4) I couldn't find a description of how to recruit participants, GFI, and online survey.

5) Are there any exclusion criteria? Do you have ethics permission? about GFI, and online survey. I don't know the relationship between qualitative analysis and questions. The question is, where does it belong to GFI?

Discussion

6)" Concerns about educational quality" is the equivalent of QS? The relationship between the title used "satisfaction", QS, and CEQ, should be explained for ease of understanding.

Why did you ask a question “how do you think ERT affects QS?”

Why didn't you extract an opinion about satisfaction from GFI?

Where did it replace, " Concerns about educational quality"

7)Is "TSP" in the table same "TSAP" in the body?　 The expression, if possible, is kind to match the body or table.
