# Supplementary material for: Perception of the quality of remote lessons in the time of COVID-19: A comparative study in Latin America quality of remote lessons in the context of COVID-19
Source: PLoS One. 2022 Jun 3;17(6):e0268966. doi: 10.1371/journal.pone.0268966 (PMC9165895; doi:10.1371/journal.pone.0268966)
Supplement: Attachment — Submitted filename: Response to Reviewers.docx [file pone.0268966.s002.docx]

**March 8, 2022**

Michael Klymkowsky, Ph.D.
Academic Editor
PLOS ONE

Dear Dr. Klymkowsky,

We would like to express our gratitude for reviewing the article titled “*Remote teaching satisfaction within the context of COVID-19: Comparative study in Latin America*”. We consider that the comments made have improved the quality of the article and we have integrated all adjustment recommendations.

Following, we provide an answer to 1) Reviewers comments, and 2) Journal Requirements.

1) **Reviewers’ comments**
1. **Introduction**

**Evaluator:** Introduction, an overview of this study is included, but there is no presentation of the purpose of the research.

**Comment**: We accept the comment. We have included the purpose of the research in the introduction.

2. **Method**

**Evaluator:**

The analysis method is reasonable. However, the method of qualitative analysis, which is the basis of this study, is unclear.

It seems that there is a lack of analytical methods for parts qualitatively.

Why did only students from the area participate in Group Focus Interview (GFI)?　How did the GIF progress?

I couldn't find a description of how to recruit participants, GFI, and online survey.

Are there any exclusion criteria? Do you have ethics permission? about GFI, and online survey. I don't know the relationship between qualitative analysis and questions. The question is, where does it belong to GFI?

**Comment**: We have described deeply the GFI, in the page 6. We post a massive invitation in the social media and participants respond to it message. All participants were voluntaries.

About the request of the area of participants, we think that the reviewer refer to the country where the GFI were applied. Effectively, GFI were performed in Colombia but, quantitative results support the similar expression of the measure in all the countries. As we show in the quantitative results, there are not differences in the construct among countries.

Finally, we clarify the relationship between qualitative analysis and quantitative questions. Qualitative findings enable to write the quantitative questions using the expressions of the participants and the dimensional structure drawn.

3. **Discussion**

**Evaluator**: 6)" Concerns about educational quality" is the equivalent of QS? The relationship between the title used "satisfaction", QS, and CEQ, should be explained for ease of understanding.

**Comment**: We have adjusted the title of the article. Moreover, the comment made by the evaluator allowed us realize the inadequate use of the term “satisfaction” which we changed for “perception”. In addition, we have eliminated QS and replaced it with “quality of lessons” which adjusts better to the research.

**Evaluator**: Why did you ask a question “how do you think ERT affects QS?” Why didn't you extract an opinion about satisfaction from GFI? Where did it replace, " Concerns about educational quality"

**Comment**: In line with the comment before, we have changed the QS concept for quality of the lessons. With the new section in the methodology, we hope to give answer to your question.

**Evaluator**: 7) Is "TSP" in the table same "TSAP" in the body?　 The expression, if possible, is kind to match the body or table.

**Comment**: We had a typography error, which we have adjusted.

**Evaluator**: Discussion can include similar studies from other countries as ERT was introduced all over the world because of COVID pandemic.

**Comment**: We included two additional studies concerning the region.

**Evaluator**: The length of the article is can be reduced by removing the repeated sentences in many paragraphs.

**Comment**: We have made a thorough review of the article, and it has allowed us to submit a shorter version.

2) **Journal requirements**

- Please ensure that your manuscript meets PLOS ONE's style requirements, including those for file naming. The PLOS ONE style templates can be found at 
  https://journals.plos.org/plosone/s/file?id=wjVg/PLOSOne_formatting_sample_main_body.pdf and 
  <https://journals.plos.org/plosone/s/file?id=ba62/PLOSOne_formatting_sample_title_authors_affiliations.pdf>

**We have reviewed and adjusted the manuscript following the indications of the files suggested.**

- We note that you have stated that you will provide repository information for your data at acceptance. Should your manuscript be accepted for publication, we will hold it until you provide the relevant accession numbers or DOIs necessary to access your data. If you wish to make changes to your Data Availability statement, please describe these changes in your cover letter and we will update your Data Availability statement to reflect the information you provide.

**The database is available and open to the public in the repository of the Universidad Cooperativa de Colombia library, and it can be consulted in the following link: <https://repository.ucc.edu.co/handle/20.500.12494/43671> or through doi: <https://doi.org/10.16925/20.500.12494/43671>**

- During our internal checks, the in-house editorial staff noted that you conducted research or obtained samples in another country. Please check the relevant national regulations and laws applying to foreign researchers and state whether you obtained the required permits and approvals. Please address this in your ethics statement in both the manuscript and submission information. In addition, please ensure that you have suitably acknowledged the contributions of any local collaborators involved in this work in your authorship list and/or Acknowledgements. Authorship criteria is based on the International Committee of Medical Journal Editors (ICMJE) Uniform Requirements for Manuscripts Submitted to Biomedical Journals - for further information please see here: <https://journals.plos.org/plosone/s/authorship>.

**In every country where data was collected, we took into consideration the ethical criteria, respecting privacy data laws, and we informed participants about the scope of the research. On the other hand, all participants answered the questionnaire online and voluntarily. In the manuscript, we have included every ethical regulation followed during the research. Furthermore, we have included the acknowledgements of the people who collaborated with the socialization of the quantitative questionnaire. Theses changes have been included in the procedure of the manuscript, and in the ethical considerations. We hope these changes solve the ethical concerns expressed by the in-house editorial staff.**

- We note that you have indicated that data from this study are available upon request. PLOS only allows data to be available upon request if there are legal or ethical restrictions on sharing data publicly. For more information on unacceptable data access restrictions, please see http://journals.plos.org/plosone/s/data-availability#loc-unacceptable-data-access-restrictions.

a) If there are ethical or legal restrictions on sharing a de-identified data set, please explain them in detail (e.g., data contain potentially sensitive information, data are owned by a third-party organization, etc.) and who has imposed them (e.g., an ethics committee). Please also provide contact information for a data access committee, ethics committee, or other institutional body to which data requests may be sent.

**The research does not have ethical or legal restrictions.**

b) If there are no restrictions, please upload the minimal anonymized data set necessary to replicate your study findings as either Supporting Information files or to a stable, public repository and provide us with the relevant URLs, DOIs, or accession numbers. For a list of acceptable repositories, please see [http://journals.plos.org/plosone/s/data-availability#loc-recommended-repositories](http://journals.plos.org/plosone/s/data-availability#loc-recommended-repositories).

**The database is available and open to the public in the repository of the Universidad Cooperativa de Colombia library, and it can be consulted in the following link: <https://repository.ucc.edu.co/handle/20.500.12494/43671> or through doi:** [**https://doi.org/10.16925/20.500.12494/43671**](https://doi.org/10.16925/20.500.12494/43671)

Thank you for your consideration!

Sincerely,

Lida Esperanza Villa Castaño

Universidad Cooperativa de Colombia, Sede Bogotá
